# Effect of AISI H13 Steel Substrate Nitriding on AlCrN, ZrN, TiSiN, and TiCrN Multilayer PVD Coatings Wear and Friction Behaviors at a Different Temperature Level

**DOI:** 10.3390/ma16041594

**Published:** 2023-02-14

**Authors:** Doğuş Özkan, Mustafa Alper Yilmaz, Deniz Karakurt, Mirosław Szala, Mariusz Walczak, Seda Ataş Bakdemir, Cenk Türküz, Egemen Sulukan

**Affiliations:** 1 Turkish Naval Academy, National Defence University, Tuzla 34942, İstanbul, Turkey; 2 Graduate School of Science and Engineering, Mechanical Engineering, Yıldız Technical University, Beşiktaş 34349, İstanbul, Turkey; 3 Barbaros Naval Science and Engineering Institute, National Defence University, Tuzla 34942, İstanbul, Turkey; 4 Department of Materials Engineering, Faculty of Mechanical Engineering, Lublin University of Technology, 20-618 Lublin, Poland; 5 Metallurgy and Materials Engineering, Marmara University, Göztepe 34722, İstanbul, Turkey; 6 Titanit Ultrahard Coatings Company, Güngören 34173, İstanbul, Turkey; 7 Maritime Faculty, Marine Engineering Department, Piri Reis University, Tuzla 34940, İstanbul, Turkey

**Keywords:** nitriding, H13 steel, arc-PVD, coatings, nanoindentation, high temperature wear, friction, tribology

## Abstract

Moving components of industrial machines and tools are subjected to wear and friction. This reduces their useful life and efficiency in running conditions, particularly at high temperatures. One of the most popular solutions is to apply an appropriate surface coating to the tribocouple’s base materials. In this study, tribometer experiments were used to evaluate the tribological performance of cathodic arc physical vapor deposited (CAPVD) AlCrN, TiSiN, CrTiN, and ZrN coatings on the gas nitrided AISI H13 tool steel to explore the effects of nitriding the steel on wear and friction behavior of these coatings at ambient and elevated temperatures. The coatings characterization is split into three main parts: mechanical, morphological, and chemical characterization. Nanoindentation has been used for mechanical characterization, thin film X-ray diffraction (XRD), and an energy-dispersive X-ray spectrometer mounted on a scanning electron microscope for chemical characterization, optical profilometer, and atomic force microscopy (AFM) for morphological characterization. Significant improvements in the adhesion qualities of the coatings to the substrate were achieved as a result of nitration. Due to this circumstance, the coatings’ load-bearing capacity and high-temperature wear resistance ratings were enhanced. The wear results showed that the AISI H13 tool steel nitriding with AlCrN and ZrN layers decreased wear rates by two to three times at 700 °C.

## 1. Introduction

AISI H13 (DIN 1.2344) hot work steel is beneficial for use in industry as die casting, extrusion dies and wear-resistant tools in terms of its high mechanical properties, thermal shock and corrosion resistance [1]. Hot work tool steels are frequently subjected to high temperatures and mechanical stresses, reducing their wear resistance. The temperature of the tool’s surface briefly rises above 500 °C because the forging temperature is typically between 950 and 1250 °C, which is higher than the tempering temperatures of standard hot work tool steel [2]. Gas nitriding is one of the surface treatment processes that allow for the formation of a hard surface layer on the substrate to improve the material’s wear and corrosion resistance [3]. The wear and corrosion resistance of AISI H13 tool steel can be enhanced to prolong the effective life of the tools at ambient and high temperatures, such as dies used in hot forming applications that are subjected to severe static/cyclic stress, erosion, thermal fatigue, and abrasive wear [4]. Under typical working conditions, hot work steels are exposed to high temperatures, resulting in substantial oxidation. Particularly high temperatures worsen the mechanical characteristics and structure of the steel, which reduces the tribological performance of AISI H13 tool steel [5]. Hard ceramic PVD coatings such as TiN, CrN, and TiAlN increase steel tools’ wear resistance, performance, and durability. Furthermore, the deposition of TiAlN coating by incorporating Al into a TiN coating enhanced the coating’s oxidation resistance, hardness, and thermal stability up to 800 °C [6,7]. TiSiN coatings were noted for being desired in high-speed and efficient machining due to their mechanical characteristics under high service conditions [8]. Numerous studies have been performed on these coatings’ high-temperature wear and friction characteristics. Altas et al. [9] used a ball-on-disc tribometer to examine the tribological characteristics of PVD-deposited AlTiN/TiSiN and TiN coatings at ambient temperature, 250 °C, and 500 °C under 2 and 5 N loads. Chang and Amrutwar [10] investigated the impact resistance of AlCrSiN, CrN, AlCrN/CrN, and AlCrSiN/AlCrN coatings produced by plasma nitriding and tested via repeated impact testing (200 k–400 k times) at ambient temperature and high temperature (500 °C). Birol et al. [11] investigated AISI H13 hot work steel coated with CrN, AlCrN, and AlTiN via a ball-on-ring tribometer at 550 °C. Sue and Chang [12] tested CrN, ZrN, and TiN-coated Inconel 718 steel substrates at 500 and 600 °C. López and Staia [13] studied the tribological behavior of magnetron-sputtered ZrN coatings at 400 and 700 °C. Wu et al. [14] investigated elevated temperature oxidation and the wear resistance of the TiAlN coating deposited on WC steel by cathodic arc evaporation and tested at 200–850 °C. These studies on high-temperature coatings showed that multi-component coatings have higher wear resistance at high temperatures. Thus, this research only investigated the tribological properties of ternary and binary coatings on nitrided or non-nitrided materials at high temperatures. However, the high-temperature tribological behavior of multilayer ceramic hard coatings has received considerable attention in the literature. Studies investigating and comparing the wear and friction behavior of multilayer and multi-component AlTiSiN and AlTiZrN diffusion layers on nitrided and non-nitrided AISI H13 steels are scant. Therefore, the nitriding effect on the high-temperature sliding behavior of multilayer cathodic arc-PVD deposited AlCrN, TiSiN, TiCrN, and ZrN coatings was explored in this research, employing ball-on-disk tribometer tests at ambient (20 °C) and high-temperature conditions (700 °C). The results confirmed a high potential of duplex treatment (nitride layer + multilayer PVD coatings) in developing the functional properties of forging dies and tools made of AISI H13 steel. 

## 2. Materials and Methods

### 2.1. Sample Preparation and Coating Process

**Figure 1:**
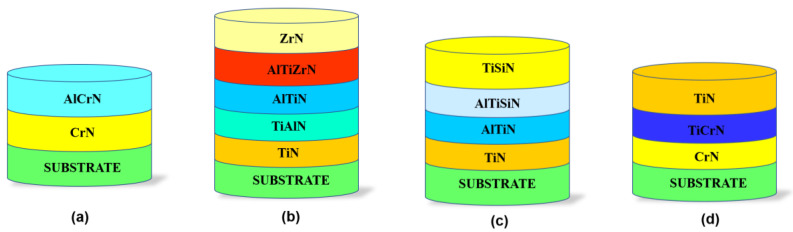
Schematic view of the coating layers architecture deposited on non-nitrided and nitride AISI H13 steel: (**a**) coating A; (**b**) coating B; (**c**) coating C and (**d**) coating D.

In coating processes, AISI H13 (X40CrMoV5-1) hot work steel discs with 62 HRC hardness, 30 mm diameter, and 5 mm thickness were used. Samples were mechanically polished to an average surface roughness of Ra = 0.07 µm using SiC grinding papers (#120–#1200 grade) and polishing cloths with 6, 3, and 1 µm diamond particles. After this, the samples were ultrasonically cleaned for 5 min in ethyl alcohol/deionized water. Applying a nitriding process to metal at 525 °C, a hardness of approx. 1250 HV can be obtained at a certain distance from the surface. Ammonia nitriding can be conducted in one or two phases before the coating process. In the first phase, high flow, low decomposition NH_3_ is delivered. In the second phase, the amount of NH_3_ is lowered for more decomposition. Two-phase penetration boosts diffusion, and so reduces penetration time. The aim of heating to 450 °C in this method is to preheat the initial step of the nitration process. Phase transformation does not occur at this temperature; it occurs at 530 °C with NH_3_ as the N source. The main objective of the nitriding process performed before the coating process is to improve the material’s surface hardness, as well as its wear resistance, fatigue life, and corrosion resistance, by minimizing the hardness decrement caused by the base materials at high temperatures. The overall nitriding cycle took around 15 h; however, the actual nitriding duration was about 6.8 h. The nitriding effect has been investigated by preparing the substrate cross-section (cut by a metallographic cutter) and the mirror polishing for metallographic microscopy evaluation. Polished samples were etched with 5% Nital for about 10–15 s. Following the nitriding process, Platit PL1001 and Interatom model Cathodic Arc Evaporation PVD systems were used to form multilayer hard ceramic coatings. The ion etching process was applied to subtract the surface by bombarding Ar gas ions at 450 °C under high bias voltage to remove impurities and to activate the substrate before the surface coating process. The four different coatings were deposited on untreated and nitrided substrates following the identical process, and each group was deposited within the unique architecture of interlayers, as shown in Figure 1. The following target materials have been used: AlTi (67/33 at.%), AlCr (65/35 at.%), and TiSi (80/20 at.%). Therefore, the coating A (CrN + AlCrN) configuration is deposited to provide superior high-temperature properties (e.g., high hardness, low friction coefficient) as well as high toughness under heavy loads or impacts [15]. AlCrN was regarded as the functional layer in the coating architecture, and CrN was chosen as the adhesive layer because it possesses lattice conformance between the steel substrate and the AlCrN structure. The coating B (TiN + AlTiN + AlTiZrN + ZrN) configuration was chosen to produce enhanced mechanical film performances at ambient temperatures, outstanding tribological properties, and desirable crystallographic phase structures at high temperatures, as stated by authors [16,17,18]. Coating C (TiN + AlTiN + AlTiSiN + TiSiN) was used to develop a nanocomposite film structure with small grain size, high hardness, high toughness, and extraordinarily high oxidation resistance at both ambient and elevated temperatures [19]. Under heavy sliding conditions, the coating D (CrN + TiCrN + TiN) configuration was selected for its high adhesion, enhanced sliding-shear resistance, and low friction coefficient against both hard and soft counterparts [20]. The coating chamber was evacuated to 5 × 10^−3^ Pa before the coating process. Coating A consisted of two layers; the first layer was the CrN adhesion layer, which was coated for 15 min using 2 pure Cr cathodes (purity 99.8%). The cathode currents were 180 A for nitrided and non-nitrided substrates. During the CrN coating process, 95 V bias was applied under 6 × 10^−3^ mbar N_2_ (purity 99.99%) partial pressure and 220 sccm flow. Following this, the second layer, AlCrN layer, was applied onto the CrN layer with 150 min coating duration, 120 A cathode current, 65 V bias voltage under 3 × 10^−2^ mbar N_2_ partial pressure, and 450 sccm flow (see Table 1). Coating B consisted of five layers. The first one was TiN, the adhesion layer, and the following layers were individual hard ceramic layers, as TiAlN/AlTiN/AlTiZrN/ZrN. Coating C was a multilayer coating consisting of a TiN adhesion layer, a second layer of AlTiN, a third layer of AlTiSiN, and a TiSiN top layer. The interlayer array of coating D was CrN/CrTiN, while the top layer was TiN. Table 1 shows all the coating process parameters, while Figure 1 shows the architecture of the coating layers.

### 2.2. Characterization of the Coatings

Before and after tribometer experiments, the surface topography of the coatings was examined using an optical microscope and atomic force microscope (AFM). Scanning electron microscope (SEM) was combined with an EDX to investigate surface tribochemistry. Raman analysis (Renishaw via reflex using a diode green laser with 50 mW power at λ = 532 nm wavelength) was performed on wear scars using a 532 nm wavelength laser beam to determine oxide structures. Wear scar cross-section and depth analyses were performed using an optical profilometer (Zeiss Smart Proof-5) and Confomap software (Digisurf). The alumina ball and coating surfaces were examined through an optical profilometer. The worn regions’ radius $\left(r\right)$, width $\left(w\right)$, and depth $\left(d\right)$ were measured to calculate wear volume according to the ASTM G99-17 by using Equations (1) and (2), respectively [21]. Five measurements in different regions of wear scar were performed, and the mean of these measurements was taken into consideration to obtain accurate wear volume.

(1) $$V_{b}=\frac{\pi \cdot d^{4}}{64\times r}$$

(2) $$V_{d}=\frac{\pi \cdot r_{wt}\cdot w_{wt}^{3}}{6r}$$

The wear rates were evaluated using Equation (3), where *F* is the normal force, *S* is the sliding distance, and *V* is the wear volume [22].

(3) $$W_{R}=\frac{V_{d}}{F\cdot S}$$

Moreover, Hertzian contact pressures were calculated by using Equation (4), where $E^{\prime }$ was the effective modulus, *F* was the normal load, and *r* was the ball radius. Table 2 shows the calculated contact pressures, which suggest that similar contact pressures and shear stresses were obtained for all the coatings.

(4) $$P_{max}=\frac{1}{\pi }{\left(\frac{6\cdot F\cdot E^{\prime 2}}{r^{2}}\right)}^{1/3}$$

(5) $$\tau_{max}=\frac{P_{max}}{3}$$

### 2.3. Tribometer Tests and Test Parameters

**Figure 2:**
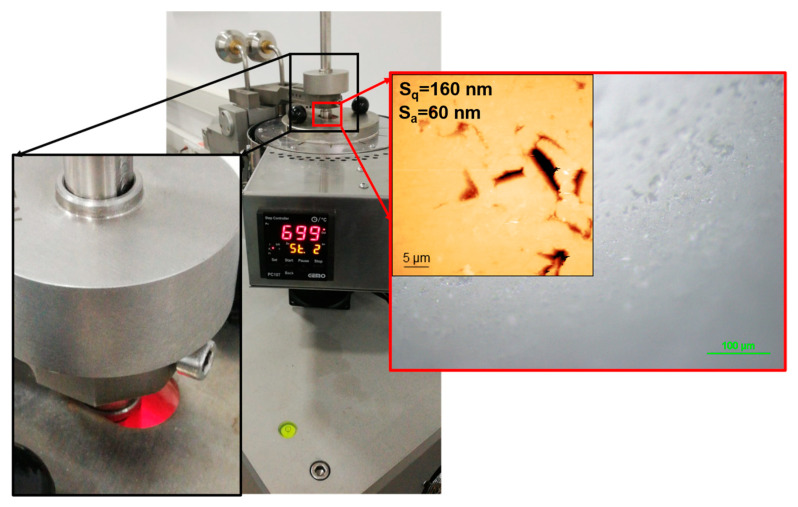
Ball-on-disk tribometer with high-temperature testing module and a scene from wear test at 700 °C with the alumina ball, optical microscope, and AFM images.

Tribometer experiments were used to explore the friction and wear characteristics of the samples at ambient temperature (20 °C) and high temperature (700 °C) with three times repetition tests. A UTS 10/20 commercial tribometer with a high-temperature pin-on-disc module was employed for friction (see Figure 1). Alumina (Al_2_O_3_) were selected as the counter surfaces for tests due to their inertness and non-reaction with the coating surface. The alumina ball had 350 GPa elastic modulus and 16 GPa hardness. The average roughness (S_a_) of 60 nm and root mean AFM measured a square roughness (Sq) of 160 nm of the ball (see Figure 2). Although the contact occurs steel-to-steel in die steels, the Al_2_O_3_ ball was chosen as the opposite surface in the study. This is explained by the fact that the Al_2_O_3_ ball does not interact with the coating at high temperatures. Thus, just the tribological properties of the coating can be evaluated better. The wear and friction tests were conducted with the same parameters for each sample. The test parameters and test conditions are shown in Table 2. The primary purpose for selecting 700 °C as the elevated testing temperature is to fit the conditions to which hot work tool steel is subjected. Therefore, the high temperature of 700 °C and loads were chosen to simulate aluminum extrusion molds and hot steel dies forging in the automotive industry, where contact pressures of 1 and 2 GPa are used. As a result, the test parameters were designed to simulate these conditions.

The Proportional Integral Derivative (PID) control system provided stable heating to keep the ambient temperature at 700 °C for the high-temperature tests. A high-temperature wear test was commonly used for surface-treated AISI H13 steel to evaluate high-temperature tribological properties [23,24]. The test duration and total sliding were 125 min, corresponding to 235 and 1099 m for 20 °C and 700 °C friction and wear tests, respectively. The purpose of choosing a different sliding distance is to ensure that the pin makes the same number of turns for each testing temperature. In other words, testing at 20 °C was conducted using a diameter of 3 mm, and testing at 700 °C temperature employed a diameter of 8 mm. For this reason, the sliding velocities are 30 and 150 m/s, respectively. In addition, the 5 N load was applied to the coating’s surface for all the experiments to obtain a Hertzian contact pressure of approximately 1.7 GPa.

**Table 2:** Tribometer test conditions of A, B, C and D coatings deposited on non-nitrided and nitrated specimens (marked as (N)).

Test Parameter	Ambient Temperature, 20 °C	Elevated Temperature, 700 °C
Load (N)	5	
Test duration (min.)	125	
Sliding distance (m)	235	1099
Temperature (°C)	20 ± 2	700 ± 2
Sliding speed (mm/s)	for 20 °C	30
	for 700 °C	150
Relative Humidity (%)	36	
Max contact pressure (GPa)		
Coating A	1.69	
Coating A(N)	1.65	
Coating B	1.80	
Coating B(N)	1.74	
Coating C	1.79	
Coating C(N)	1.75	
Coating D	1.67	
Coating D(N)	1.73	
Shear stress (GPa)		
Coating A	0.56	
Coating A(N)	0.55	
Coating B	0.58	
Coating B(N)	0.56	
Coating C	0.59	
Coating C(N)	0.57	
Coating D	0.54	
Coating D(N)	0.56	

Crystal structure, grain size, strain, and phase analyses were performed using an X-ray diffractometer with the CuKα radiation (*λ* = 1.54056 Å). The coatings’ crystallite size (*D*) was calculated using Equation (5), known as the Scherer equation, in which *K* is the Sherrer constant, *λ* is the wavelength of the X-ray source, and *β* is full width at half maximum of the XRD peaks [25].

(6) $$D=\frac{K\cdot \lambda }{\beta \cdot \cos\theta }$$

The lattice strain (*ε*) was evaluated by using Equation (6)

(7) $$\varepsilon =\frac{\beta \cos\theta }{4\sin\theta }$$

## 3. Results

### 3.1. Morphological Characterization and Mechanical Properties

**Figure 3:**
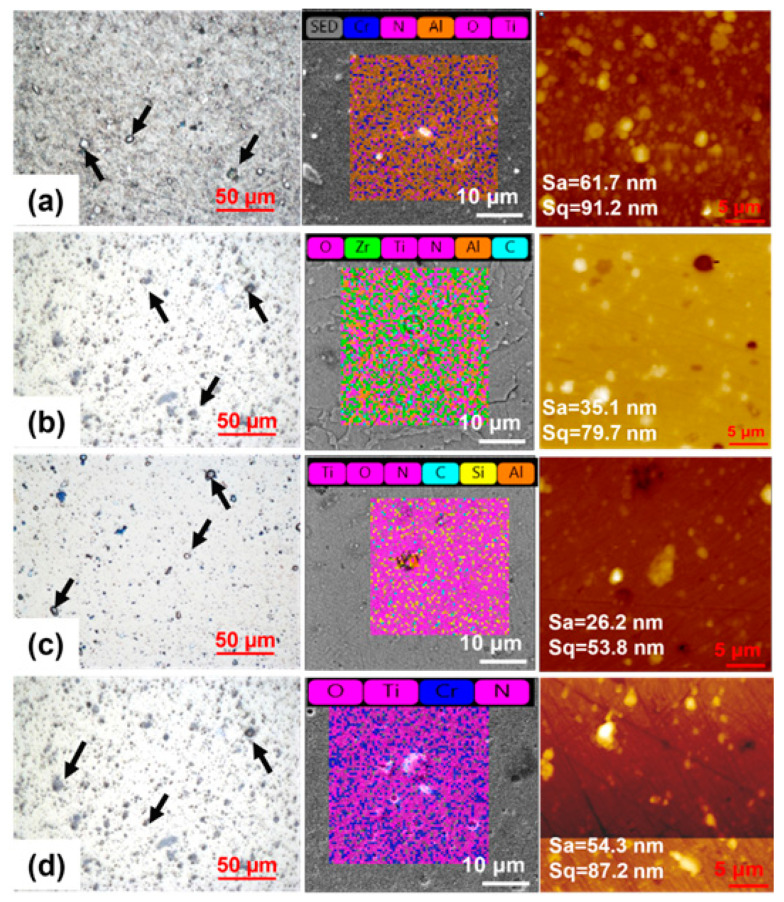
Optical microscope, AFM, and SEM/EDX analysis of (**a**) coating A, (**b**) coating B, (**c**) coating C, and (**d**) coating D; droplets are marked by arrows.

The optical microscope, AFM, and SEM/EDX images showed the surface of the coating after deposition (see Figure 3). The AFM measurement was conducted using lateral force microscopy. Images from an optical microscope and AFM exhibit droplets of molten cathode material (see black arrows in Figure 3) on the surfaces of the coatings. These droplets were higher in coatings C and D than in coatings A and B. Cr:Al:N was discovered in atomic concentrations of 13.0:23.0:51.2 percent for coating A, and these elements were determined to be 14.1:23.2:49.9 on the surface of coating A(N). A similar Cr/Al stoichiometric of 0.56/0.60 was obtained for coating A. Ti:Si:N ratios were found to be 40.5:8.8:45 for coating C(N), and were found to be 40.9:8.0:44.1 for coating C(N), with a Si/Ti stoichiometry of 0.22/0.20. When examining the coating D surface, Ti:Cr:N was determined to be 17.0:5.0:24.4 in at.%, while Ti:Cr:N for coating D(N) was 19.3:6.2:27.5. Ti:Zr:Al:N elements were confirmed to be 8.3:8.5:5.5:16.6 in atomic percent for coating B, whereas they were found to be 9.1:8.8:5.2:15.4 on the coating B(N) surface. AFM topography examinations explored the coatings’ average surface roughness. The measured S_a_/S_q_ values ranged from 61.7 nm/91.2 nm, 35.1 nm/79.7 nm, 26.2 nm/53.8 nm, and 54.3 nm/87.2 nm for A, B, C, and D coatings, respectively.

**Figure 4:**
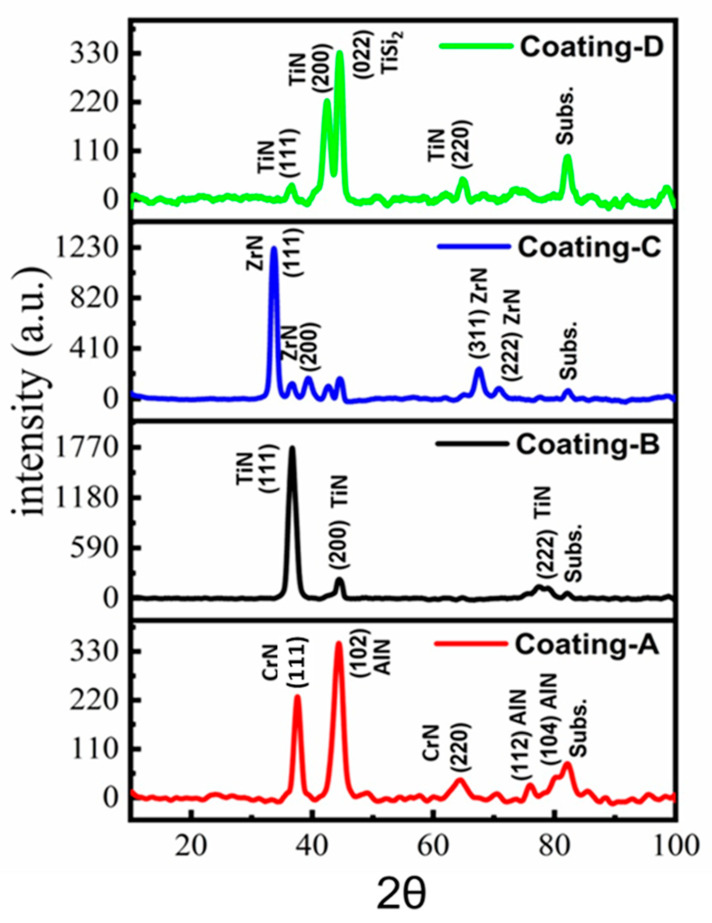
Phase analysis of the coatings, XRD.

The XRD patterns of the coatings are shown in Figure 4. In the XRD profile of the coatings, A indicates CrN(111), AlN(102), CrN(220), AlN(112) and AlN(104) [26,27]. The dominant orientation of hexagonal crystal structure AlN(102) was a grain size of 5.8 nm, and for CrN(111) it was a grain size of 6.3 nm. For Al_1−x_Cr_x_N, if the X value is between 0.29 and 0.69, it is stated that the structure of Al_1−x_Cr_x_N is fcc B1 solid solution. However, hexagonal AlN can be formed in coatings containing rich Cr and Al by a spinodal mechanism. In coating B, peaks at 36.82°, 44.46°, and 82.17° correspond to TiN(111), TiN(200), and CrN(222) phases, respectively. The preferred orientation was cubic TiN(111) with a grain size of 8.4 nm [28,29]. Detected 2θ angles at 33.67°, 39.37°, 44.57°, and 67.56° corresponded to ZrN(111), ZrN(200), ZrN(311), and ZrN(222), and the dominant orientation was cubic ZrN(111) with the 8.6 nm grain size for coating C, respectively [30]. Furthermore, peaks at 36.38°, 42.25°, 44.54°, and 64.88° can be attributed to TiN(111), TiN(200), TiSi2(022), and TiN(220) for the coating D, respectively [31,32,33]. The preferred orientation was face-centered TiSi_2_(022), and the average grain size was determined to be 7.5 nm. Strain calculations revealed that the lowest crystal lattice deformation was found for coating D, whereas the highest lattice deformation was obtained for coating B (see Table 2). 

**Figure 5:**
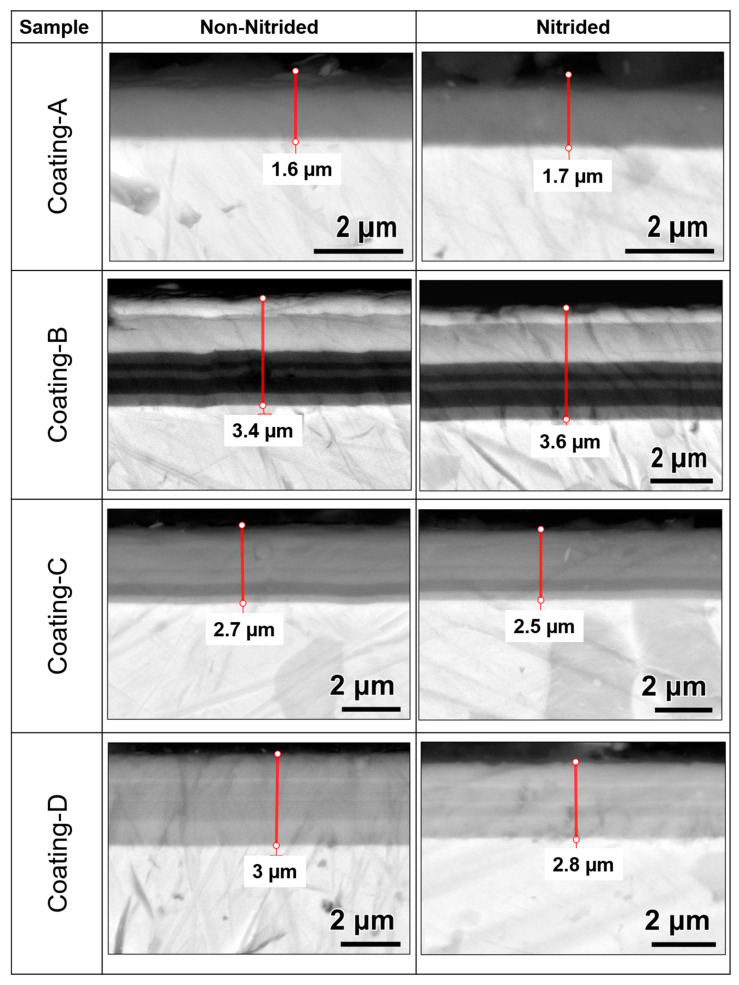
SEM cross-section images of the coatings.

Coatings thickness was measured in the cross-sectional samples view shown in Figure 5. Coating thickness changed from 1.6 µm to 3.6 µm where the color changes indicated multilayers for coatings B, C, and D. A nano-indenter was used to evaluate hardness and Young’s modulus of coatings at certain points of coating indent depth (max. 10% of coating thickness). Nano hardness measurements revealed that coating B had the highest hardness, 49.2 and 48.71 GPa on nitride and non-nitride steel surfaces, respectively, whereas coating C and coating D(N) had the lowest hardnesses of 31.61 and 33 GPa, respectively. In the general comparison of hardness results, it can be seen that non-nitrided samples have higher hardness than nitrated samples. This trend is because nitrided coatings cause deviations in results with higher roughness values, as seen in Table 2 [34]. The considerable delamination around the scar in both coatings suggests that the coatings’ adhesion was poor, resulting in low hardness. Under stress, the covering fractures readily and cannot withstand plastic deformation. It was observed that all coating hardness and elastic modulus decreased on nitrided steels (see Table 3). H/E ratio was calculated to elastic strain to failure and indicated the toughness property of the coatings, while H^3^/E^2^ set out resistance to plastic deformation [33,35]. Based on the H/E and H^3^/E^2^ ratios, coating B demonstrated the best toughness property and resistance to deformation in the range of 0.1/0.5 and 0.1/0.5 on nitride and non-nitrided steel, respectively. Steel nitration generally decreased the H^3^/E^2^ ratios of the coatings; however, an increase in the H^3^/E^2^ ratio from 0.2 to 0.3 was found in coatings C and C(N).

**Figure 6:**
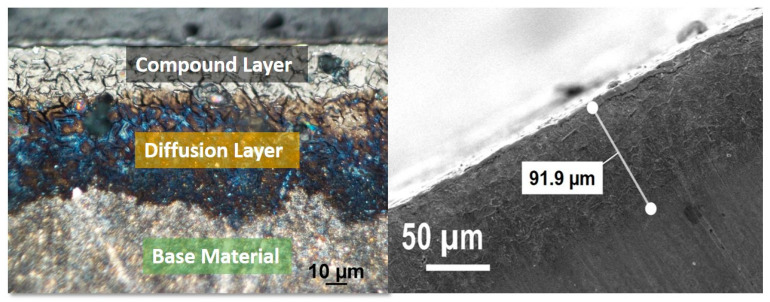
Optical microscope and SEM cross-section images of the nitrided layer.

**Figure 7:**
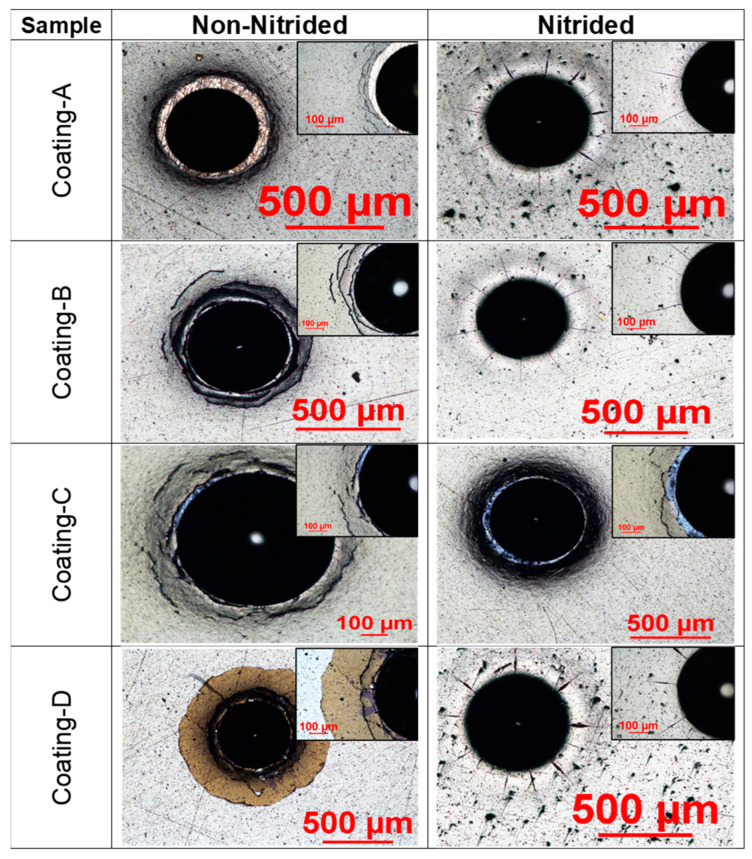
Results of adhesion testing, according to the ISO 26443:2008 standard.

The nitrided layer on steel was investigated by optical microscope and SEM, which was shown in Figure 6. The average thickness of the measured diffusion nitration layer was 91.9 µm, which is comparable to the depth of the nitrided layer reported by the literature for K340 tool steel [27]. The adhesion of the coatings was investigated using Rockwell adhesion testing relating to indentations, as described in ISO26443:2008 [36]. Furthermore, 150 kgf was chosen as a higher load that penetrates up to the base material for coatings of similar thickness in the literature. The primary objective of this investigation was to determine the adhesion of the coatings to the steel substrate. The optical microscope was used to examine the indentation tracks to obtain the adhesion strength of the coating using the Daimler-Benz scale, which ranges from HF1 to HF6 [37]. The Rockwell imprints are classified as HF1 and HF2, corresponding to adequate adhesion and accepted adhesion level for the coatings [38]. Coating A on non-nitride steel demonstrated HF4 level adhesion in Figure 7, whereas coating A on nitrided steel demonstrated HF-1 level adhesion. This suggested that nitrided steel had better adhesion to coating A and lowered internal stress. Coating B exhibited HF-5 level adhesion on non-nitrided steel with high delamination, while coating B had HF-1 level adhesion on nitrided steel with just a few cracks [39,40]. Furthermore, coating C demonstrated weaker adhesion on non-nitrided steel and nitrided steel, with higher delamination, labelled as HF-5 and HF-4, respectively. 

On the other hand, coating D non-nitrided steel had an HF-6 level that exhibited more delamination than coating C, with the lowest adhesion. Coating D on non-nitrided steel, on the other side, showed good adhesion at the HF-1 level. Adhesion tests revealed disparities in adhesion behavior among identical coating designs, which the nitration effect could explain. While applying TiN or CrN adhesion layers, nitrided steel boosted the adhesion of ions and atoms on the surface. As a result, TiN and CrN layers used to provide surface adherence for upper layers demonstrated better adhesion on nitrided steel than on non-nitrided steel [41].

### 3.2. Friction and Wear Properties

**Figure 8:**
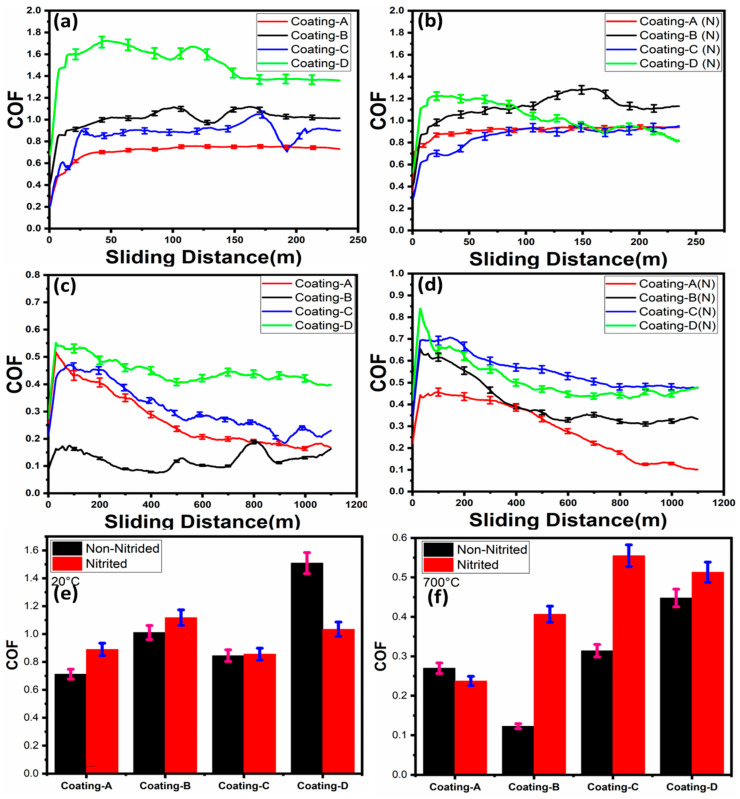
Friction coefficients of the coatings: (**a**) at ambient temperature (20 °C) on non-nitrided steel, (**b**) at ambient temperature (20 °C) on nitrided steel, (**c**) at elevated temperature (700 °C) on non-nitrided steel, (**d**) at elevated temperature (700 °C) on nitrided steel, (**e**) average COFs at 20 °C, (**f**) average COFs at 700 °C.

The coatings’ average COFs were obtained by plotting vs. total sliding distance at 20 °C and 700 °C, as shown in Figure 8. Results suggested the lowest and steady-state COF of 0.75 for coating A. In contrast, coating D had the highest average COF of 1.4 at ambient temperature because of the lowest hardness and undesired adhesion level of Coating D. The COF of coating D decreased from 1.6 to 1.3, which was a steady-state regime at 150 m due to the degradation of the TiN top layer (see Figure 8a). After the degradation of the TiN top layer, the TiCrN layer established TiO_2_ and Cr_2_O_3_ oxides which decreased the COF value. The COF of coatings B and C were found to be 0.9 and 1, with observed fluctuations in the COF curves, respectively. Coatings A and B showed similar COFs on nitrided steel: around 0.8. However, contrary to non-nitrided steel, the coating D COF slightly decreased from 1.2 to 1 at 90 m due to the degradation of the top layer, and then COF decreased to 0.8. Furthermore, on both non-nitrided and nitrided steel, coating B had an average COF of 0.9 (see Figure 8b). In the elevated temperature tests, coating B exhibited the lowest COF of 0.17 near the steady-state regime on non-nitrided steel (see Figure 8c). Coating A’s COF progressively decreased from 0.5 to 0.2 until it reached a sliding distance of 500 m. Then, a steady-state friction regime was observed. Coating D on non-nitrided steel exhibited the highest COF, around 0.5. When the COFs of the coatings deposited on nitrided steel were examined, a substantial reduction in friction from 0.4 to 0.1 was noted for coating A after 300 m of sliding distance at 700 °C (see Figure 8d). Additionally, the COF of coating B decreased from 0.6 to 0.4 until a sliding distance of 400 m was reached, resulting in a steady-state friction regime. After 500 m of sliding distance, coating C achieved a steady-state friction regime. Friction reduction and steady-state friction regimes could be linked to the formation of elevated temperature oxide layers. In addition, the wear of the roughness peaks leads to a decrease in contact pressures, resulting in a decrease in friction. The lack of steady-state COF in coating D was primarily linked to the glazing layer formation. Although the glazing layer was commonly thought to be a protective layer, as layer thickness grows it loses its protective characteristic and promotes three-body abrasive wear. 

Even if the nanostructured layers have enhanced mechanical characteristics at room temperature, these layers change as the temperature rises due to recovery, recrystallization, and grain development processes. [42]. The average COFs of the tribometer tests with three repetitions were shown in Figure 8e,f. Coating C had lower COFs on nitrided or non-nitrided steel than the other coatings, whereas coating D had the highest average COF of approximately 1.5 at 20 °C. However, coating B showed the lowest COF on non-nitrided steel, with an average COF of 0.12 at 700 °C (see Figure 8f). 

### 3.3. Wear Rates of the Coatings

**Figure 9:**
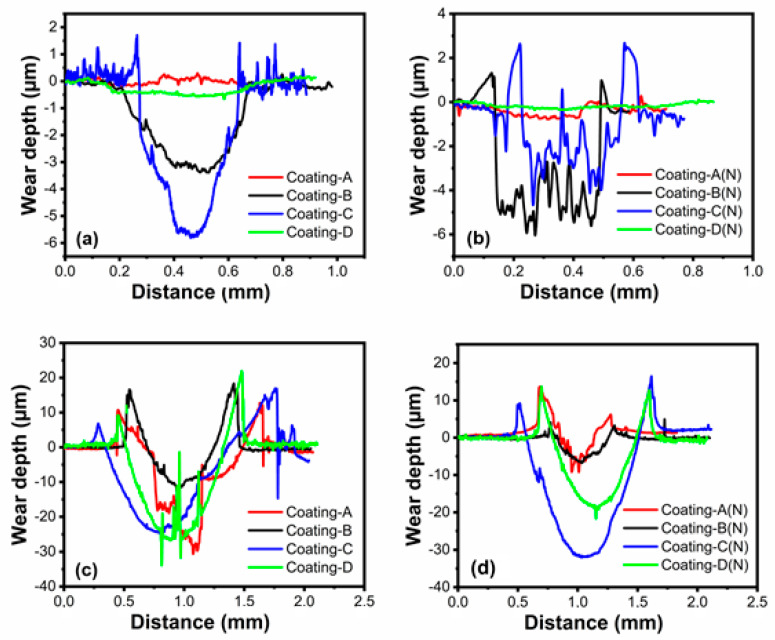
Wear scar profiles of the coatings: (**a**) wear scar depth profiles of the coatings on non-nitrided steel at 20 °C, (**b**) wear depth profiles of the coatings on nitrided steel at 20 °C, (**c**) wear scar depth profiles of the coatings on non-nitrided steel at 700 °C, (**d**) wear scar depth profiles of the coatings on nitrided steel at 700 °C.

Wear depth profiles of the coatings were evaluated by optical profilometer measurements after wear tests (see Figure 9). A comparison of the coatings’ wear depths tested at 20 °C revealed the best wear resistance, not only for coatings A and C, but also for coatings A(N) and C(N) (see Figure 9a,b). In addition, the deepest wear scar was obtained for coating D, with approximately 6 µm, whereas coating B revealed a 3.5 µm wear scar depth. 

**Figure 10:**
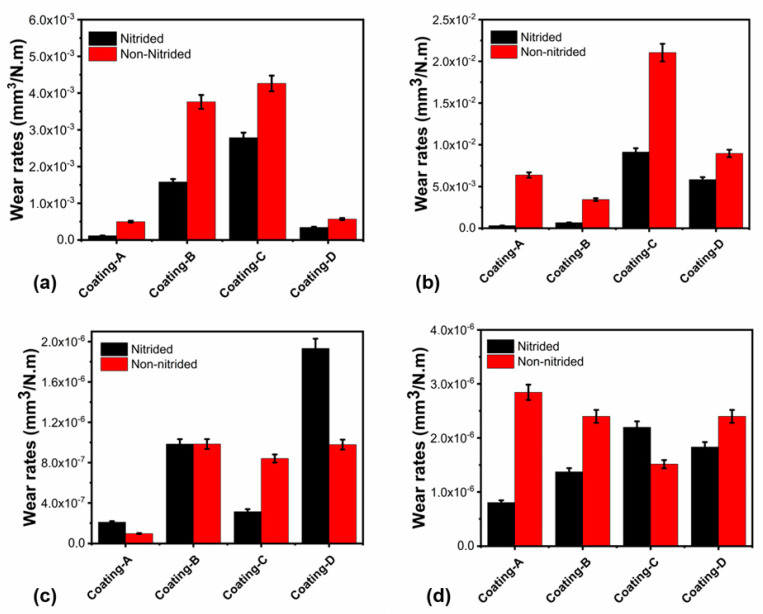
(**a**) Wear rates of the coatings at 20 °C, (**b**) wear rates of the coatings at 700 °C, (**c**) wear rates of the ball at 20 °C, (**d**) wear rates of the ball at 700 °C.

**Figure 11:**
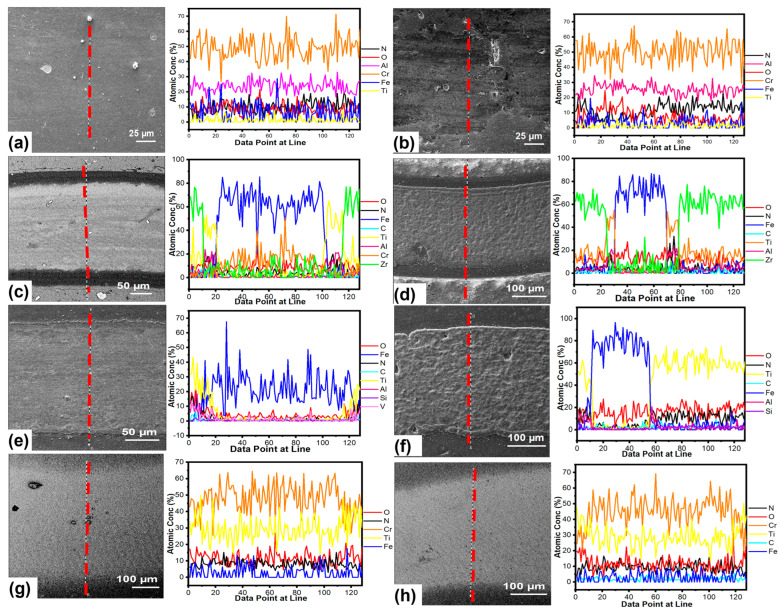
SEM/EDX lines scans of the worn surfaces at ambient temperature wear and friction test; (**a**,**b**) coating A on non-nitrided steel and nitrided steel, (**c**,**d**) coating B on non-nitrided steel and nitrided steel, (**e**,**f**) coating C on non-nitrided steel and nitrided steel, (**g**,**h**) coating D on non-nitrided steel and nitrided steel, respectively.

Furthermore, the wear resistance of coatings A and C on nitrided steel (see Figure 9b) was similar to non-nitrided steel due to the similar wear depth (see Figure 9a). On the other hand, coating D’s lower wear scar depth on the nitrided steel demonstrated enhanced wear resistance compared to non-nitrided steel at 20 °C, which follows with the wear rates shown in Figure 10. Furthermore, the line EDX measurement Figure 11d derived from the wear trace indicates that the coating is not removed from the surface. While Ti and Cr signals are strongly received on the surface, the iron signal originating from the base material may be observed in other coatings with entirely worn surfaces. Additionally, strong narrow valleys suggest abrasive wear tracks, which were seen at 20 °C for coating B and D on nitrided steel rather than non-nitrided steel. Both wear scar profiles and the wear rate of coatings at 700 °C showed the best wear resistance for coating B both on nitrided and non-nitrided steel with the lowest wear scar depth. However, as compared to non-nitrided steel, nitriding the steel sample enhanced the anti-wear performance of coating B (AlTiZrN+ZrN coating) at an elevated temperature of 700 °C. Although coating C demonstrated excellent anti-wear performance in ambient temperature wear testing, it had a poor anti-wear performance at 700 °C. These significant differences in wear resistance at two temperatures can be derived from the dropping of the hardness by about 60% after high temperature wear tests, as seen in Table 4. Figure 10 shows the wear rates of the coatings and counter surface balls, which are consistent with the wear scar width and depth measurement findings. Coating B demonstrated comparable wear resistance at 20 °C on both nitrided and non-nitrided steels, with wear rates of 3.95 × 10^−3^ and 1.62 × 10^−3^ mm^3^/Nm, respectively. According to the wear rate examination, coating A demonstrated the best wear resistance at ambient temperature on both nitrided and non-nitrided steel, with wear rates of 1.16 × 10^−4^ and 4.96 × 10^−4^ mm^3^/Nm, respectively. When compared to non-nitrided steel, the nitration process increased the wear resistance of the CrN/AlCrN layers by approximately five times due to improved adhesion behavior to the steel substrate and load-bearing capacity. According to the literature review, coating wear resistance is closely linked to coating adhesion to the substrate. Many duplex techniques aim to improve coating adhesion and the load-bearing capacity of coated surfaces by depositing an interlayer material before coating or surface treatment, such as nitriding [43]. Coatings formed on nitrided AISI H13 steel grow preferentially on the diffusion zone, mainly comprised of dissolved nitrogen atoms, alloying elements in a solid solution, and dispersed fine nitrides in the metal. The diffusion layer improves coating–substrate adhesion, which leads to easier nucleation in this phase. This layer’s rough surface promotes greater adhesion between the steel and the coating [44]. The mechanical locking theory of Pizzi and Mittal can be used to clarify this issue. According to this theory, rough surfaces have more irregularities in roughness tips, grooves, or holes than smoother surfaces, resulting in improved substrate–coating adhesion. On the other hand, the covalent bond between the coating and stable nitrides has bonding energy much higher than steel’s metallic bond [45,46]. Furthermore, the highest wear rate of 4.36 × 10^−3^ mm^3^/Nm was recorded for coating C on nitrided steel. On the other hand, coating C exhibited a wear rate of 2.92 × 10^−3^ mm^3^/Nm on non-nitrided steel. H^3^/E^2^ ratios can explain the wear resistance variation for coating C, which decreased to 0.2 on nitrided steel. Further, coating C with an HF-5 level adhesion and a lower H3/E2 ratio in ambient temperature testing showed weaker plastic deformation resistance than other coatings. Moreover, nitration of the steel boosted coating D’s wear resistance by around two times during ambient temperature wear testing (see Figure 10a). According to high-temperature friction and wear testing, coating A had the best wear resistance on nitrided steel, with a wear rate of 3.13 × 10^−4^ mm^3^/Nm. Nitriding the steel substrate, in particular, showed remarkable wear resistance enhancement for coating A, with the wear rate decreasing from 6.37 × 10^−3^ mm^3^/Nm to 3.13 × 10^−4^ mm^3^/Nm (see Figure 10b). Coating B, on the other hand, was the second most wear-resistant coating with a wear rate of 6.63 × 10^−4^ mm^3^/Nm, whilst coating C had the lowest wear resistance on both nitrided and non-nitrided steels. Moreover, the third wear-resistant coating was coating D, both on nitrided and non-nitrided steel, with wear rates of 5.82 × 10^−3^ mm^3^/Nm and 8.96 × 10^−3^ mm^3^/Nm, respectively. Finally, nitration to steel improved the wear resistance of multilayer AlCrN, ZrN, and TiSiN coatings for high-temperature applications.

The ball surface wear rates were consistent with the coating wear rates, as shown in Figure 10c,d. An increase in the wear rate of the coating’s surface decreased the wear rate of the ball, and vice versa.

### 3.4. Chemical Analysis of the Worn Surfaces

SEM/EDX line scan analyses were performed on the wear scar, and EDX line scans confirmed the wear scar depth and wear rate results. Figure 11 displays SEM images and EDX line scans of worn surfaces that were tested at room temperature. According to the line scans, the presence of Cr and Al without Fe on the surface indicated that there was no substantial wear on the surface for both coatings A on non-nitrided and nitrided steel (see Figure 11a,b).

**Figure 12:**
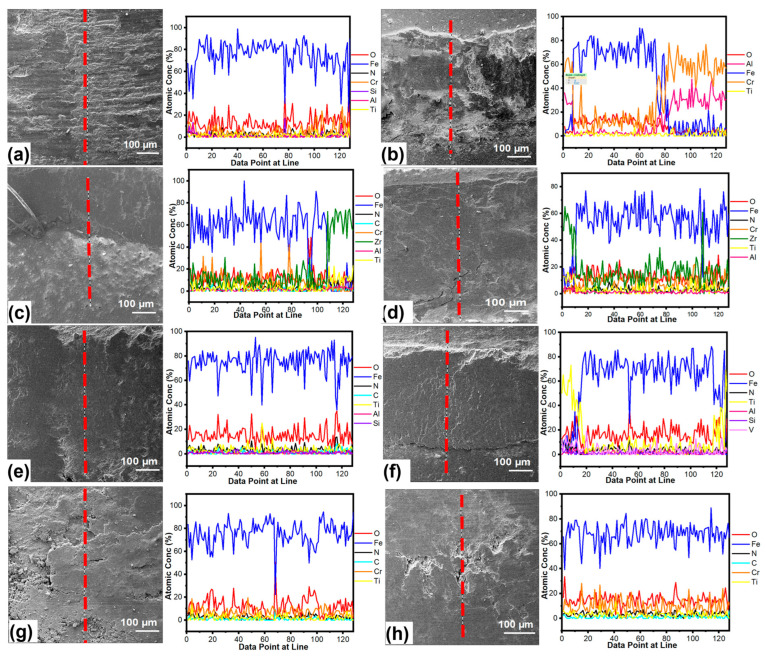
SEM/EDX lines scans of the worn surfaces at 700 °C wear and friction test; (**a**,**b**) coating A on non-nitrided steel and nitrided steel, (**c**,**d**) coating B on non-nitrided steel and nitrided steel, (**e**,**f**) coating C on non-nitrided steel and nitrided steel, (**g**,**h**) coating D on non-nitrided steel and nitrided steel, respectively.

Additionally, wear scar depth measurements verified the wear resistance of coating A, suggesting that the AlCrN coating functioned well during ambient tribometer experiments. The EDX scan line of the wear scar, on the other hand, presented a higher level of wear for coatings B and C owing to Fe detection (see Figure 11c–f). These findings demonstrated that coatings B and C were removed and worn to the base material, as seen by wear scar depth measurements in Figure 10a,b. Only the first TiN layer was removed, and the TiCrN layer was identified by finding Cr and Ti elements in the wear scar of coating D (see Figure 11g,h). SEM images and an EDX line scan for the 700 °C tests indicated that the coatings had completely worn down to the underlying material, with Fe detected on wear scar surfaces (see Figure 12). Furthermore, more plastic deformation and coating degradation was found for coatings. When compared to other coatings, coating D on non-nitrided steel showed more plastic deformation and degradation (see Figure 12g). On the other hand, delamination and plastic deformation were higher in coating A when compared to coatings B and C (see Figure 12b).

**Figure 13:**
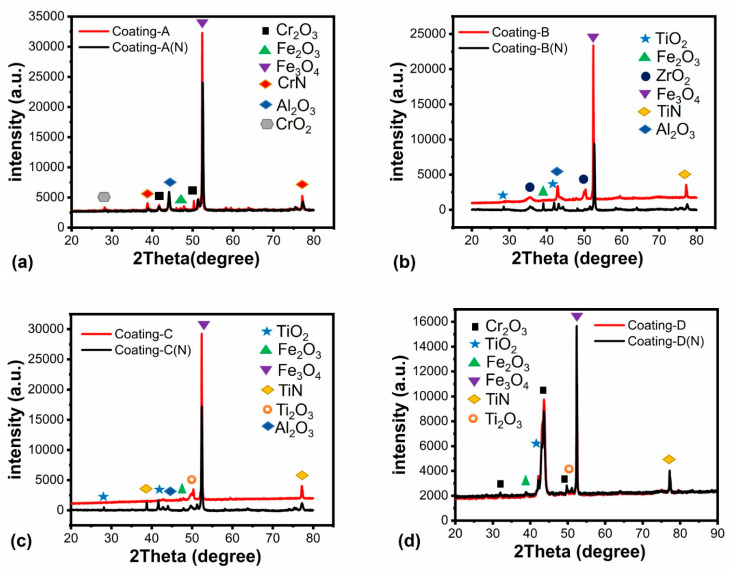
Phase analysis of the coatings after elevated temperature tests, XRD: (**a**) coating A; (**b**) coating B; (**c**) coating C and (**d**) coating D.

To investigate the development of crystal structure, the XRD studies were performed on the surfaces tested at 700 °C. According to XRD investigations, coatings were significantly oxidized during higher temperature wear testing. Dissolving the N in TiN caused substantial oxidation in coating D, whose initial TiN layer oxidized and produced Ti_2_O_2_ and TiO_2_. Furthermore, the TiCrN second layer oxidized, resulting in TiO_2_ and Cr_2_O_3_ crystal orientation (see Figure 13d) [47,48,49]. Although Fe_3_O_4_ was the dominating phase, the TiN(420) phase was identified at 77.2° [50]. Similarly, the oxidation of coating A formed CrO_2_, Al_2_O_3_, and Cr_2_O_3_^,^ where the Fe_2_O_3_ and Fe_3_O_4_ structure originated from the wear scar surface (see Figure 13a) [51,52]. The CrN(311) phase was detected at 77.2° for coating A on both non-nitrided and nitride steel surfaces.

**Figure 14:**
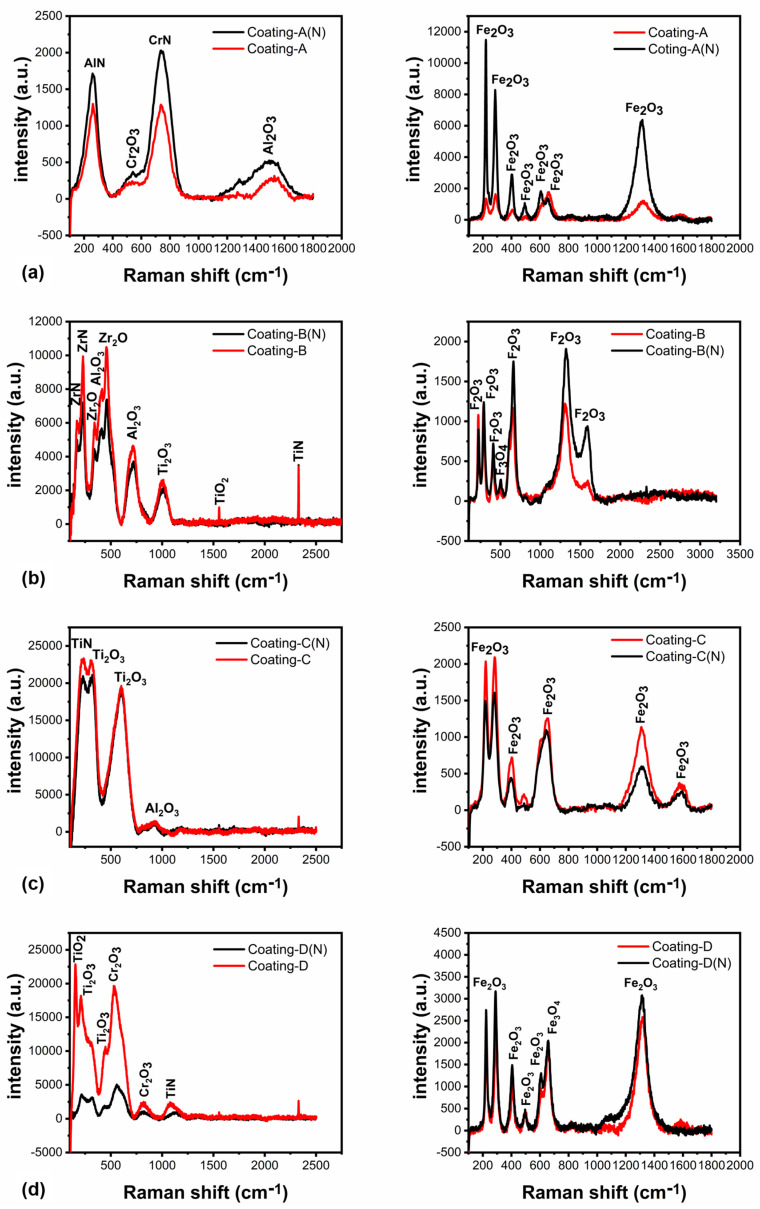
Raman analysis of the unworn and wear scar surfaces of the coatings tested at 700 °C: (**a**) coating A; (**b**) coating B; (**c**) coating C and (**d**) coating D.

The oxidation of ZrN and AlTiZrN layers produced Al_2_O_3_, ZrO_2_, and TiO_2_ crystal structures in coating B at elevated temperatures (see Figure 13b) [53]. Moreover, the oxidation of AlTiSiN+TiSiN layers carried out Ti_2_O_3_, Al_2_O_3_, and TiO_2_ structures as illustrated in XRD spectra in Figure 13c. Following the 700 °C testing, Raman spectra of the coatings’ unworn and wear scar surfaces were acquired in addition to XRD analyses (see Figure 14). The face-centered cubic AlN peak was obtained at 260.9 cm^−1^, and the CrN peak was found at 746 cm^−1^ (see Figure 14a) on the unworn surface of the coating A. Coating A also oxidized and formed Cr_2_O_3_ and Al_2_O_3_ on the unworn surfaces, which were detected at 540 and 1496 cm^−1^. It is consistent with the XRD results given in [54]. Furthermore, iron oxides in the form of α-Fe_2_O_3_ were found on the wear scar surface of coating A, indicating that the base material was reached at the end of the 700 °C tests [55]. The oxidation of the top ZrN and the second layer of AlTiZrN produced ZrO_2_ (342 and 460 cm^−1^), Al_2_O_3_ (416 and 716 cm^−1^), and TiO_2_ (1002 and 1555 cm^−1^) crystal structures for coating B [56,57]. Similar to coating A, iron oxides were detected on the wear scar surface, setting out the base material reached (see Figure 14b). The peaks at 302 and 600 cm^−1^ were assigned to TiO_2,_ and peaks at 925 cm^−1^ could be attributed to Al_2_O_3,_ which were detected on the coating C surface due to TiSiN and sub-layers oxidation; see Figure 14c [56]. Raman peaks at 156 cm^−1^ were attributed to TiO_2_, whereas peaks at 210 and 447 were assigned to Ti_2_O_3_. Peaks at 528 and 813 cm^−1^, on the other hand, could be attributed to Cr_2_O_3_, which was formed as a consequence of the TiCrN layer oxidation (see Figure 14d) [58]. Similarly, Raman analysis of the wear scar surfaces showed Fe_2_O_3_ and Fe_3_O_4_ peaks originating from the base material steel [59].

After 700 °C tests, the coatings’ hardness deviation was assessed using nanoindentation tests (see Table 5). Coatings A and B on nitrided steels had the lowest hardness deviations of 25.8 and 25.4%, respectively, whereas coating C on non-nitrided steel had the highest hardness variation of 60.5%. The hardness comparison showed that deviations were consistent with the wear rates, highlighting the reason for the coatings wear resistance.

## 4. Discussion

Multilayer coatings were subjected to ambient and high-temperature wear and friction tests to investigate their wear resistance, particularly at 700 °C. Cathodic arc PVD was used to produce AlCrN, ZrN, TiSiN, and TiN coatings with ceramic composite interlayers and thicknesses ranging from 1.6 to 3.6 µm on nitrided and non-nitrided AISI H13 tool steels. Wear rates at room temperature demonstrated that coatings A and D had better wear resistance. The high hardness and elastic modulus of CrN+AlCrN and TiCrN+TiN layers can explain the wear resistance of these coatings. On the other hand, these coatings exhibited comparable toughness with E/H ratios, while H^3^/E^2^ ratios offered superior anti-wear performance to coatings B and C. Mo et al. [60] utilized multiple arc-PVD to deposit CrN and AlCrN coatings on cemented carbide steel, and found 32.5 GPa hardness and 474.80 GPa elastic modulus for the AlCrN coating, which is similar to our findings. Furthermore, nitriding steel reduced the hardness and elastic modulus of these coatings, as well as the H^3^/E^2^ ratios, resulting in higher wear resistance as compared to coatings deposited on non-nitrided steel. These coatings were tested at high temperatures over long distances to determine their wear resistance for molding and turbine blade coating applications and to investigate nitriding steel’s impact on the wear and friction of these coatings. 

According to the wear rate comparison, coatings A and B exhibited good anti-wear performance at 700 °C. Coatings C and D, on the other hand, had poor wear resistance, which may be explained by the oxidation degree of the coating surfaces. The TiN layer, whose oxidation limit was reported to be 600 °C, caused a higher level of coating degradation and delamination in coating D [61]. When this temperature is exceeded, TiN converts to TiO_2_ by releasing N during the oxidation process, as demonstrated in the XRD spectra in Figure 12, which were obtained following experiments [62]. Otani and Hoffman [63] found that the sublayer TiCrN oxidized to form a TiO_2_ outer layer and a Cr_2_O_3_ inner layer as a TiO_2_ + Cr_2_O_3_ mixture. This mixture’s average grain sizes were 20.4 nm for TiO_2_ and 28.3 nm for Cr_2_O_3_. The Al_2_O_3_ + Cr_2_O_3_ mixture resulted from the oxidation of AlCrN in coating A, which had higher wear resistance than coating D. The typical grain sizes of this mixture were 19 nm for Al_2_O_3_ and 11.9 nm for Cr_2_O_3_. Concerning the coating D wear rate, the lower wear resistance could be attributed to the Cr_2_O_3_ phase, which decreases the wear strength of the coating structure and is dominant in the oxidation process of the TiN and TiCrN layers. Cr_2_O_3_ exhibited lower thermal conductivity than Al_2_O_3_ and TiO_2_ phases. Thus, it could not dissipate heat efficiently, resulting in higher local temperatures and a reduction in the hardness of the TiCrN layer during 700 °C wear tests. The Cr_2_O_3_ phase, on the other hand, was inhibited by the Al_2_O_3_ phase during the oxidation process, and the Al_2_O_3_ phase also stabilized the Cr_2_O_3_ phase, preventing higher hardness in the AlCrN coating structure [64]. It was discovered that the Cr_2_O_3_ phase, combined with smaller grain sizes, improved the coating structure’s wear resistance by avoiding coating delamination and degradation. Avoiding bigger grain size Cr_2_O_3_ with the help of the Al_2_O_3_ phase revealed the best anti-wear performance for coating on H13 steel. Structures with finer grain size have a longer grain boundary length, and therefore oxidation kinetics are different and more prone to oxidation compared to coarse-grained structures. Since grain boundaries are unstable regions, oxygen creates a pathway for diffusion and these high-energy regions at the surface could make sliding more difficult and increase the friction force of materials [65]. Stott and others [66] have indicated that wear-protective layers are produced by compaction and sintering of oxidized wear debris particles on the rubbing surfaces, and the compact oxide layers are beneficial to the decrease in friction and wear. As the oxide grain size decreases, the oxygen diffusion coefficient and the sintering rate of the oxide particles increases, and the tribofilm formation rate increases. The wear resistance of the coating increases in direct proportion to the rate of tribofilm formation [67]. The oxidation of ZrN and AlTiZrN layers, on the other hand, formed TiO_2_, ZrO_2_, and Al_2_O_3_ phases with average grain sizes of 45 nm, 23.8 nm, and 23.2 nm, respectively. For coating B, these oxide structures offered extremely good wear resistance on both non-nitrided and nitrided steel. This can be explained by the Al_2_O_3_ and ZrO_2_’s high thermal conductivity, which alleviated the hardness reduction of the AlTiZrN layer by efficiently enabling heat dissipation [68]. Furthermore, the Raman study confirmed XRD findings of oxidation in the coatings’ top and sub-layers. Additionally, the nitriding process formed an approx. 1300 HV compound layer on the steel surface, which improved mechanical strength and served as a thermal barrier, preventing hardness loss. The thermal barrier effect is related to the formation of a nitride layer, which has a lower thermal conductivity than the sample and is formed during the nitriding process [69]. An increase in parameters such as temperature and time directly affects the thickness of the nitration layer, and the thermal conductivity decreases by increasing the thickness of the nitride layer. In addition, the thermal diffusion value, defined as the amount of heat energy spread per unit of time, also decreased with the increase of nitride layer thickness [70,71]. Therefore, following the opinion given in the work [72], it can be concluded that the nitrided layer significantly increases the thermal stability of the coatings sublayers and prevents hardness decrease, as shown in Table 4 and Table 5, and affects resistance to abrasion and plastic deformation at 700 °C.

## 5. Conclusions 

Multilayer coatings were deposited on non-nitrided and nitrided AISI H13 steel substrates using a cathodic arc PVD system. Coatings were examined for wear friction and behaviors at ambient and elevated temperatures. The mechanical and morphological characteristics of the coatings were also investigated. When the Rockwell C adhesion findings are examined, it is clear that the nitration process enhanced the adhesion greatly, particularly for coating D. Cracks and separations in the coating are decreased as a result of this increase in both the load-carrying capability and the adhesion quality of the nitration process, and therefore the COF decreased. Coatings A and D had the highest wear resistance, owing to their high hardness and elastic modulus in ambient temperature wear and friction tests. In addition, coatings A and B demonstrated the best wear resistance at 700 °C, which the TiO_2_, ZrO_2,_ could explain, and Al_2_O_3_ phase forms were observed in the XRD and Raman data. These oxide layers reduced the thermal conductivity of the coatings, preventing a rapid reduction in hardness and stabilizing the coating’s mechanical characteristics at 700 °C. Nitriding the steel and producing a hundred-micron diffusion layer slowed the hardness loss of the coatings by providing a thermal barrier between the steel and the coatings.

As a result, coatings deposited on nitrided steels outperformed coatings deposited on non-nitrided steels in terms of wear resistance. Furthermore, based on highlighted findings, coatings A and B had similar and lowest hardness deviations of 25% after 700 °C tests, resulting in improved wear resistance for high temperature applications such as molding.

## Figures and Tables

**Table 1:** Coating parameters of the AlCrN, ZrN, TiSiN, and TiCrN.

Coating Array	Duration (Min.)	Coating	Cathode Type and Quantity	Cathode Current (A)	Bias Voltage (V)	Power (W)	Temperature (°C)	Gas Flow sccm	Nitrogen Partial Pressure (mbar)
Coating A	15 min.	CrN (1 µm)	2 Cr	180	95	17,100	450	220	6 × 10^−3^
	150 min.	AlCrN (0.6 µm)	2 AlCr	120	65	7800	450	450	3 × 10^−2^
Coating B	45 min.	TiN (0.3 µm)	1 Ti	125	95	11,875	450	220	6 × 10^−3^
	30 min	TiAlN (0.5 µm)	1 Ti 1 AlTi	120 Ti 120 AlTi	65	7800	450	350	2.5 × 10^−2^
	25 min.	AlTiN (0.5 µm)	2 AlTi	150	65	14,250	450	400	2.5 × 10^−2^
	25 min.	AlTiZrN (1.5 µm)	2 AlTi 1 Zr	120 AlTi 180 Zr	95	11,400 17,100	450	450	2.5 × 10^−2^
	30 min.	ZrN (0.8 µm)	1 Zr	200	120	2400	450	400	2.5 × 10^−2^
Coating C	15 min.	TiN (0.3 µm)	1 Ti	125	95	11,875	450	220	6 × 10^−3^
	40 min.	AlTiN (0.5 µm)	2 AlTi	150	65	14,250	450	400	2.5 × 10^−2^
	70 min.	AlTiSiN (0.5 µm)	1 Ti 2 AlTi 1 TiSi	120 TiSi 150 AlTi	65	7800 14,250	450	480	2.5 × 10^−2^
	95 min.	TiSiN (1.2 µm)	1 TiSi	150	65	15,250	450	480	3.2 × 10^−2^
Coating D	45 min.	CrN (1 µm)	4 Cr	70	120	8400	380	250	2 × 10^−2^
	60 min.	TiCrN (1 µm)	4 Cr 4 Ti	70 Cr 60 Ti	120	8400 7200	380	300	2 × 10^−2^
	15 min.	TiN (1 µm)	4 Ti	60	100	6000	380	200	1.5 × 10^−2^

**Table 3:** Mechanical and structural properties of the coatings.

Coating Sample	Hardness (GPa)	Elastic Modulus (GPa)	H/E H^3^/E^2^ (GPa)	S_a_/S_q_ (nm)	Coating Thickness (µm)	Preferred Orientation Grain Size (nm)	Strain (ε)
Coating A	42.45	438.46	0.1 0.4	61.7/ 91.2	1.6	5.8	2.2
Coating A(N)	35.60	401.28	0.1 0.3		1.7		
Coating B	49.23	490.1	0.1 0.5	35.1/ 79.7	3.4	8.4	5.6
Coating B(N)	48.71	479.07	0.1 0.5		3.6		
Coating C	42.77	460.40	0.1 0.3	26.2/ 53.8	2.7	8.6	4.7
Coating C(N)	31.61	415.57	0.1 0.2		2.5		
Coating D	43.65	534.56	0.1 0.3	54.3/ 87.2	3.0	7.5	1.1
Coating D(N)	33.00	464.47	0.1 0.2		2.8		

**Table 4:** Wear rate and wear depth of coatings.

Coating Sample	Wear Rate 20 °C	Wear Rate 700 °C	Ball Wear Rate 20 °C	Ball Wear Rate 700 °C	Wear Depth 20 °C	Wear Depth 700 °C
Coating A	4.97 × 10^−4^	6.37 × 10^−3^	9.62 × 10^−8^	2.84 × 10^−6^	0.65	18.19
Coating A(N)	4.97 × 10^−4^	3.13 × 10^−4^	2.09 × 10^−7^	8.03 × 10^−7^	0.17	7.6
Coating B	3.95 × 10^−3^	3.43 × 10^−3^	9.83 × 10^−7^	2.39 × 10^−6^	3.20	12.51
Coating B(N)	1.62 × 10^−3^	6.63 × 10^−4^	9.83 × 10^−7^	1.37 × 10^−6^	4.45	6.67
Coating C	4.36 × 10^−3^	2.10 × 10^−2^	8.00 × 10^−7^	1.51 × 10^−6^	5.72	24.60
Coating C(N)	2.92 × 10^−3^	9.13 × 10^−3^	3.13 × 10^−7^	2.19 × 10^−6^	3.12	32.27
Coating D	6.87 × 10^−4^	8.96 × 10^−3^	9.78 × 10^−7^	2.39 × 10^−6^	0.52	26.34
Coating D(N)	1.12 × 10^−3^	5.83 × 10^−3^	1.93 × 10^−6^	1.83 × 10^−6^	0.37	18.35

**Table 5:** Hardness comparison of the coatings before and after 700 °C tests.

Coating Sample	Hardness (GPa) (Before 700 °C Wear Test)	Hardness (GPa) (After 700 °C Wear Test)	Deviation (%)
Coating A	42.5	26.4	43.8
Coating A(N)	35.6	23.9	25.8
Coating B	49.2	32.6	34.9
Coating B(N)	48.7	36.3	25.4
Coating C	42.8	16.9	60.5
Coating C(N)	31.6	24.4	38.6
Coating D	43.7	19.6	55.1
Coating D(N)	33	17.2	47.8

## Data Availability

Data is contained within the article.

## Abbreviations

AFM	Atomic force microscope
COF	Coefficient of friction
EDX	Energy dispersive X-ray
PID	Proportional integral derivative
PVD	Physical vapor deposition
SEM	Scanning electron microscope
SPIP	Scanning probe image processor
XRD	X-ray diffraction
**Nomenclature**	
*R*	Diameter of the ball (mm)
*R*	Radius of the ball (mm)
*d*	Worn area diameter of the ball (mm)
*r_wt_*	Wear track radius (mm)
*w_wt_*	Wear track width (mm)
*V_b_*	Volume losses of ball (mm^3^)
*V_d_*	Volume losses of disk (mm^3^)
*W_R_*	Wear rate (mm^3^/N m)
*H*	Hardness (GPa)
*E*	Elastic modulus (GPa)
*E*′	Effective elastic moduli (GPa)
*F*	Normal load (N)
*P_maks_*	Maximum contact pressure (GPa)
*R_a_*	Arithmetic mean roughness of coatings (nm)
*S_a_*	Surface average roughness (nm)
*S*	Sliding distance (m)
*U*	Sliding speed (m/s)
*μm*	Length (μm)
*D*	The crystallite sizes (nm)
*K*	Sherrer constant
*β*	Full-width at half maximum of XRD peaks
*λ*	Wavelength of the X-ray source (nm)
*θ*	Angle of diffraction peak (radian)
*ε*	Lattice strain
*H*^3^/*E*^2^	Modulation ratio
